# Protein Trafficking through the Endosomal System Prepares Intracellular Parasites for a Home Invasion

**DOI:** 10.1371/journal.ppat.1003629

**Published:** 2013-10-24

**Authors:** Stanislas Tomavo, Christian Slomianny, Markus Meissner, Vern B. Carruthers

**Affiliations:** 1 Center for Infection and Immunity of Lille, CNRS UMR 8204, INSERM U 1019, Institut Pasteur de Lille, Université Lille Nord de France, Lille, France; 2 Laboratory of Cell Physiology, INSERM U 1003, Université Lille Nord de France, Villeneuve d'Ascq, Lille, France; 3 Wellcome Trust Centre for Molecular Parasitology, Institute of Infection, Immunity and Inflammation, University of Glasgow, Glasgow, United Kingdom; 4 Department of Microbiology and Immunology, University of Michigan Medical School, Ann Arbor, Michigan, United States of America; International Centre for Genetic Engineering and Biotechnology, India

## Abstract

*Toxoplasma* (toxoplasmosis) and *Plasmodium* (malaria) use unique secretory organelles for migration, cell invasion, manipulation of host cell functions, and cell egress. In particular, the apical secretory micronemes and rhoptries of apicomplexan parasites are essential for successful host infection. New findings reveal that the contents of these organelles, which are transported through the endoplasmic reticulum (ER) and Golgi, also require the parasite endosome-like system to access their respective organelles. In this review, we discuss recent findings that demonstrate that these parasites reduced their endosomal system and modified classical regulators of this pathway for the biogenesis of apical organelles.

The phylum Apicomplexa is comprised of single-celled parasites of eminent clinical and economic importance including *Plasmodium* spp., the agents of malaria, and *Toxoplasma gondii*, the cause of toxoplasmosis. *P. falciparum* is the most notorious member of the Apicomplexa. Each year, 300–500 million people suffer from *falciparum* malaria while about 1 million individuals, mostly children, succumb to the infection [1]. *T. gondii* has long been recognized as a congenital pathogen, and the advent of AIDS focused attention on this apicomplexan parasite as a life-threatening opportunistic pathogen [2]. The phylum Apicomplexa also includes other notable members that infect humans and animals such as *Cryptosporidium*, a cause of acute gastrointestinal disease, and *Eimeria*, a major cause of poultry disease.

*Toxoplasma* and *Plasmodium* are the most experimentally tractable among apicomplexan parasites because they can be cultured *in vitro*, have experimental rodent models, and are amenable to genetic manipulation. *Toxoplasma* is distinguished from nearly all other members of the phylum Apicomplexa by its ability to infect virtually all warm-blooded animals and humans, replicating in many different nucleated cell types therein. The versatility of *T. gondii* facilitates its use as a model system not only for shared aspects of its pathogenic kin including the malaria parasite, but also more widely for intracellular parasitism and infection biology. *Plasmodium* on the other hand is much more selective in its host cell range, exclusively infecting red blood cell (RBCs) for the pathogenic phase of its life cycle. Despite their differences, both parasites are remarkably successful in their ability to avoid immune elimination. While these parasites use a variety of immune evasion strategies, cell invasion and intracellular residence are key tactics that are shared by virtually all apicomplexan parasites. Specialized regulated secretory organelles termed micronemes and rhoptries are crucial to cell invasion and intracellular survival of most apicomplexan parasites.

Micronemes, which are small ovoid secretory organelles, ensure the timely calcium-dependent release of adhesive proteins involved in host cell recognition and entry by the parasite (Figure 1A and 1B). Microneme proteins, termed MICs in *Toxoplasma* and various names in *Plasmodium*, also contribute to parasite gliding motility and egress from host cells after intracellular replication [3], [4]. Rhoptries are club-shaped secretory organelles that consist of a neck portion containing RON proteins (termed RONs in many apicomplexans) and a bulbous section containing proteins (termed ROPs in *T. gondii*) (Figure 1A and 1B) [5]. Rhoptry discharge during invasion delivers RONs into the host cell cytoplasm and plasma membrane where a subset of these proteins is used as parasite-derived receptors in the moving junction, a ring-like structure through which the parasite invades the host cell [6], [7]. ROPs, which are also injected into the host cell cytoplasm, function in *T. gondii* to manipulate host pathways including those that drive innate immunity [8], [9]. Several *Plasmodium* rhoptry proteins contribute to parasite binding and invasion of host cells [10]. Interestingly, *P. berghei* ookinetes do not have rhoptries and yet still invade host cells [11]. In addition, *Theileria* sporozoites release their rhoptry contents post invasion [12]. Thus, the striking association of rhoptries with host cell invasion is not universal among apicomplexans. Nevertheless, the crucial functions of micronemes and rhoptries depend on the correct targeting to and packaging of MICs, RONs, and ROPs into these secretory organelles. Whereas considerable progress has been made in understanding the general and specific functions of micronemes and rhoptries, less was known until recently about the cellular machinery and trafficking routes involved in their biogenesis.

**Figure 1:**
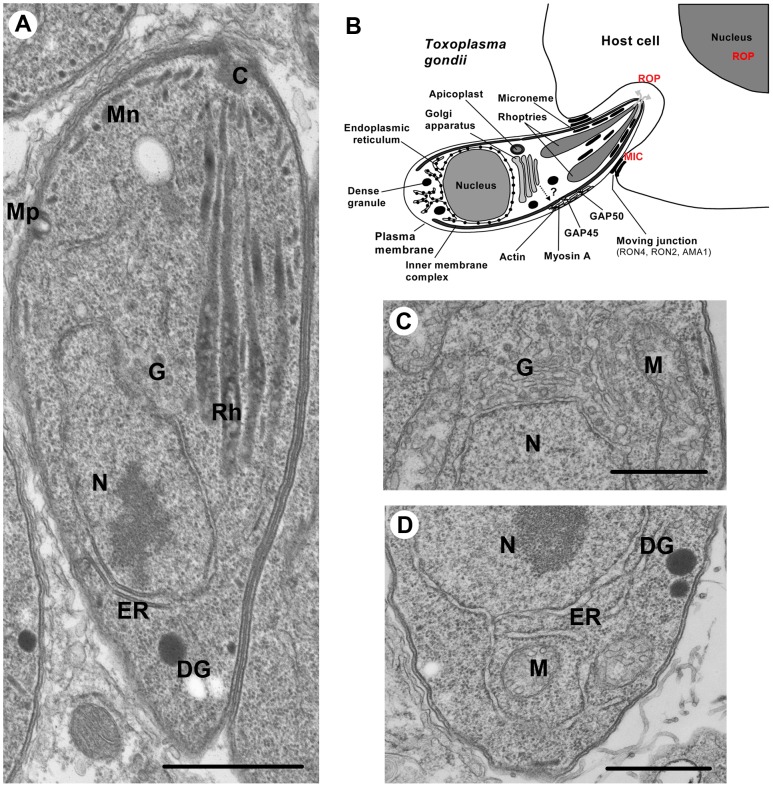
Ultrastructure of *Toxoplasma gondii*. (**A**) Intracellular *T. gondii* tachyzoite showing the MICs (Mn), ROPs (Rh), micropore (Mp), Golgi (G), nucleus (N), endoplasmic reticulum (ER), and dense granules (DG). (**B**) A schematic picture of *T. gondii* entering into a nucleated mammalian host cell. The apical exocytosis of MICs deploys onto the parasite surface MIC proteins required for parasite motility and the formation of moving junction. ROP secretion provides the ROP proteins that are involved in host cell invasion and modulation of immune responses. The constitutive secretion of dense granules (DG) is involved in the modification of the parasitophorous vacuole (PV). (**C**) Higher magnification of the single Golgi apparatus of *T. gondii*. (**D**) Higher magnification of the *T. gondii* ER. Scale bars, 1 µm.

This review will focus on recent insight into the trafficking of proteins to micronemes and rhoptries based mainly on studies of *T. gondii*; however, where appropriate, reference is also made to malaria parasites.

## The Secretory Pathway Is Highly Polarized in Apicomplexan Parasites

Eukaryotic cells have a highly complex endomembrane system that is largely conserved across all phyla and includes the ER, Golgi, and major exocytic pathways [13]. Intriguingly, recent comparative genome analysis provided compelling evidence that the endomembrane system of the last common eukaryotic ancestor was very complex with most trafficking factors present. For example, it appears that the last common eukaryotic ancestor possessed as many as 23 Rab-GTPases, which is a significantly greater repertoire than that of many modern organisms and indicates a major role for secondary loss in the evolutionary diversification of eukaryotic endomembrane [14]. Indeed, for apicomplexans, between nine (*Theileria*) and 15 (*Toxoplasma*) Rab-GTPases have been identified [15].

Nevertheless, apicomplexans have an endomembrane system that is both simple and elaborate. By virtue of their small size and obligate parasitic lifestyle, apicomplexans are stripped-down examples of the eukaryotic design, allowing an appreciation of the minimal equipment necessary for secretion. For example, with dimensions of ∼2×7 µm, *T. gondii* is substantially smaller than a typical mammalian cell. Visualization by electron microscopy reveals an elaborate endomembranous system including a single nucleus, mitochondrion, a plastid-like organelle termed the apicoplast, an interconnected ER network, a single stacked Golgi apparatus, the inner membrane complex (IMC), and the apical secretory organelles (Figure 1B, 1C, and 1D). Whereas all secretory proteins appear to traffic through the ER and Golgi, thereafter they faithfully target to their separate destinations in the micronemes, rhoptries, and a third type of secretory organelle termed dense granules. The ability to visualize and reconstruct the entire polarized secretory pathway at high resolution provides the means to test theories regarding the mechanisms of transport through the ER, Golgi, and subsequent radiation to the secretory organelles [16], [17].

One of the most appealing aspects of the *T. gondii* system is the parasite's unique mode of replication by endodyogeny, whereby two daughter cells are newly born within the mother. The nucleus is centrally located, essentially bisecting the rapidly dividing tachyzoites. The ER, although distributed throughout the zoite, is more concentrated posterior to the nucleus (Figure 1D). In contrast to a mammalian cell, the *T. gondii* ER is so reduced that the nuclear envelope itself provides a substantial proportion of its total volume [18]. Electron microscopy reveals thinly coated vesicles budding from the anterior transitional ER, destined for the closely juxtaposed single Golgi stack (Figure 1C). Similar observations have been made in *Plasmodium* except that the Golgi is a vesicular structure [19]. Whereas in mammalian cells hundreds of Golgi stacks occupy the perinuclear area [20], the Golgi apparatus of most apicomplexans consists of a single structure of three to five cisternae [21]. The relative simplicity of the *T. gondii* Golgi was elegantly exploited as a model to address Golgi biogenesis and segregation in eukaryotes [22]. Beyond this common depot, aspects of the formation and transport of MICs and RONs/ROPs in the parasite apical end have been described for *T. gondii* tachyzoites [23]–[25], and *Plasmodium* sporozoites [19] and merozoites [26], [27]. It should be noted though that most of these studies were essentially morphological descriptions and mechanisms underlying vesicular trafficking to apical organelles have remained largely hypothetical until recently.

## Trafficking Proteins through the ER-Golgi to Secretory Organelles in Apicomplexan Parasites

Apicomplexan parasites target proteins to dense granules, micronemes, and rhoptries using both conserved and unusual mechanisms [28]–[30]. Dense granules are the default pathway for proteins devoid of specific forward targeting information. Although no ultrastructural studies of dense granule formation have been reported, it is presumed that the biogenesis of these secretory organelles occurs at the Golgi, the site of dense-core granule formation in most mammalian cells. This conjecture is based in part on the absence of proteolytic maturation for dense granule proteins, a process that is associated with post-Golgi compartments.

On the other hand, evidence is mounting in *T. gondii* that trafficking to the micronemes involves not only the Golgi but also a post-Golgi system that is endosome-like. The term “endosome-like” is used because although putative endosomal vesicles have been identified based on markers (e.g., rab5, rab7, and sortilin) that are typically associated with the endosomal system, classical endocytosis has not been validated in *T. gondii* (see also below). The endosomal system of most eukaryotes is used for the uptake and processing of surface components and exogenous material, which is endocytosed into early endosomes (EE) that mature into late endosomes (LE) before fusing with lysosomes. This system is usually distinct from the late secretory system, which includes regulated secretory organelles. A series of studies suggested that MICs transit the endosome-like system, based on the findings that immature pro-MICs are seen in structures bearing late endosomal markers (LE) [30], [31] and that at least one MIC accumulates in late LE upon mutation of targeting elements contained in its pro-peptide [32]. Additionally, nascent micronemes were documented in close proximity to an endolysosomal compartment (the vacuolar compartment/plant-like vacuole) marked with a cathepsin L protease [33]. This protease was also reported to act as a maturase for at least two MICs [34], lending further support to MIC trafficking through the endosomal system. In *Plasmodium*, the participation of the endosomal system in protein secretion is less clear since electron microscopy observations suggested that micronemes and rhoptries form directly from the Golgi apparatus [19], [26], [27]. However, organellar markers were not available to identify the specific sites of biogenesis, thus additional studies are necessary to examine the potential involvement of the endosomal system.

**Figure 2:**
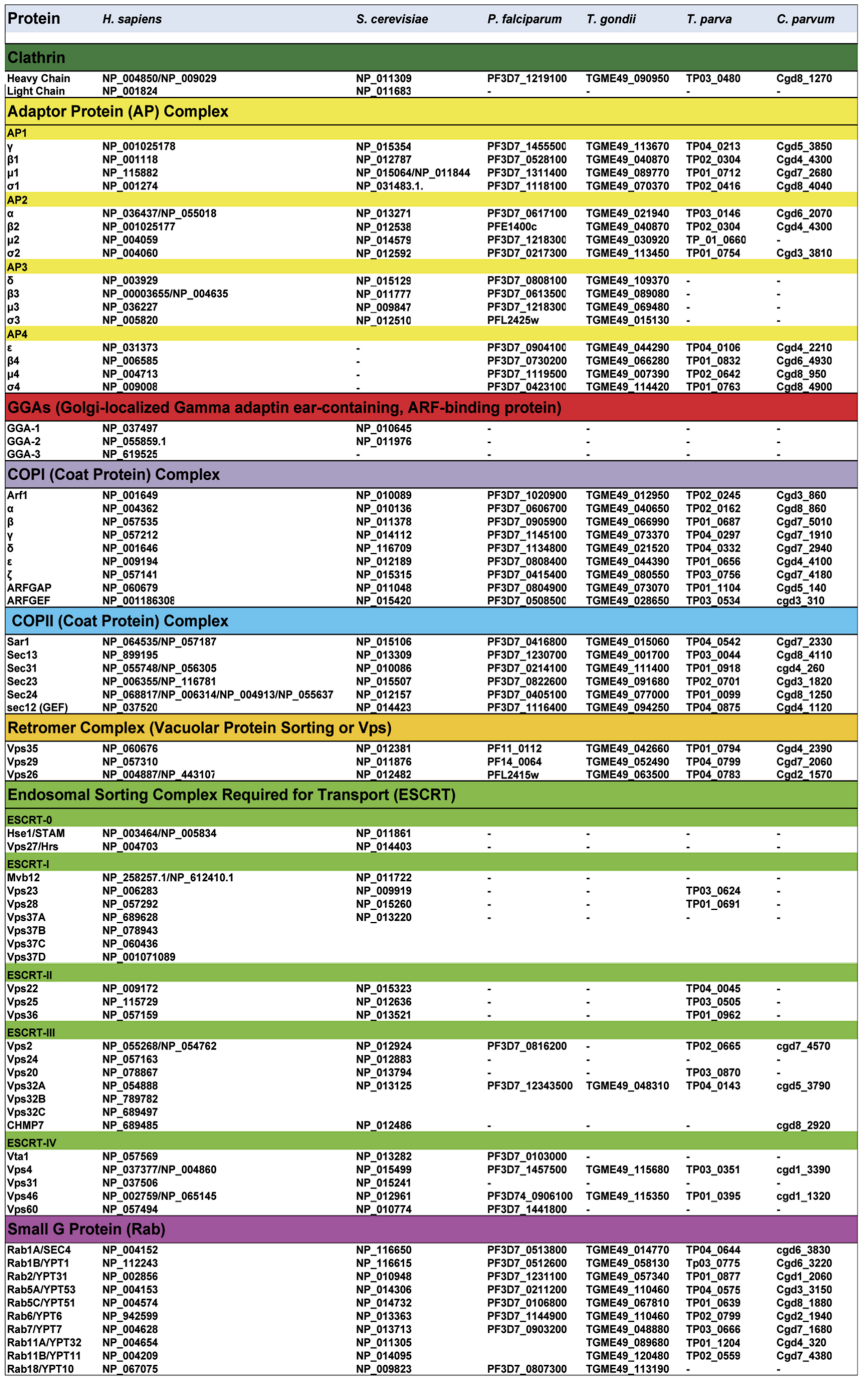
Comparative bioinformatics analysis of genes coding components of vesicle-mediated trafficking and endosomal sorting in apicomplexan parasites, *Saccharomyces cerevisiae*, and *Homo sapiens*. Most of these genes and their corresponding accession numbers were collected from Eupathdb.org (for apicomplexan parasites) and uniprot.org (yeast and human cells). The data from apicomplexan parasites *Toxoplasma gondii* (*T. gondii*), *Plasmodium falciparum* (*P. falciparum*), *Theileria parva* (*T. parva*), and *Cryptosporidium parvum* (*C. parvum*) were compared with human (*H. sapiens*) and the yeast *Saccharomyces cerevisiae* (*S. cerevisiae*). AP, adaptor protein; GGAs, Golgi-localized, γ-ear–containing, ADP-ribosylation factor binding protein; COPI, Coatomer complex I (retrograde transport from trans-Golgi apparatus to cis-Golgi and endoplasmic reticulum); COPII, Coatomer complex II (anterograde transport from ER to the cis-Golgi); ESCRT, Endosomal Sorting Complex Required for Transport.

*T. gondii* acidic vesicular structures that form transiently in the apical region just prior to cytokinesis are thought to be precursors of rhoptries [35]. Similar to the situation in micronemes, an ROP mutant defective in transport to the rhoptries accumulated in putative endosomal structures identified by expression of an ATPase mutant of Vps4 [36]. In other systems, this Vps mutant is a well-documented marker of multivesicular bodies, a type of LE that contains internal vesicles. Interestingly, Vps4 is one of the few members of the ESCRT protein family identified in the genomes of apicomplexan parasites (Figure 2). ESCRT proteins are best known for their role in forming the internally budding vesicles seen in multivesicular bodies. Proteolytic maturation of ROPs likely occurs in the acidic pre-rhoptries prior to their development into mature rhoptries with segregated bulbous and neck regions. Recent reports suggest that distinct cues within the proteins are necessary for segregation into the bulbous and neck regions [37]. Although rhoptry protein maturation in *T. gondii* was initially thought to involve a cathepsin B protease [38], targeted disruption of this enzyme failed to alter ROP maturation or rhoptry biogenesis [39]. Overall, whereas dense granule formation likely occurs at the Golgi, the biogenesis of micronemes and rhoptries is distinguished by the involvement of the endosome-like system in *T. gondii* and possibly other apicomplexans. Although this situation is unusual as compared to regulated secretory organelles in many cell types, it is not unique since certain mammalian blood cells including natural killer cells and platelets contain regulated secretory granules that are derived from the endolysosomal system [40].

Specific targeting elements within MICs and ROPs have been identified and implicated in vesicular transport to the respective organelles. Micronemes contain both soluble and transmembrane proteins, and correct targeting of soluble MICs requires their association with membrane-anchored partners [41], [42]. Two tyrosine-based motifs, SYHYY (conforming to the YXXØ motif where X is any residue and Ø is a hydrophobic residue) and EIEYE, in the cytoplasmic tail of one MIC were shown to be critical for sorting to the micronemes when the tail was fused to a heterologous protein. It was also noted that putative tyrosine-based sorting signals are present in the cytoplasmic tails of several MICs, implying that these motifs function similar to higher eukaryotes by binding adaptor subunits to facilitate post-Golgi clathrin-coated vesicle formation [41], [42]. While these findings remain to be validated with MICs in a more natural state, many of the classical components of vesicular trafficking are present in the genome of *P. falciparum and T. gondii* (Figure 2), supporting the notion of sorting via recognition of cytoplasmic motifs. Studies in *Plasmodium* suggest that, like micronemes, sorting to the rhoptries involves the formation of multi-protein complexes [43]. This work showed that rhoptry targeting of soluble protein complexes (RAP1-3 or PfRhopH1-3) requires their association with a glycosyl-phosphatidyl inositol-anchored and lipid raft-associated protein, rhoptry-associated membrane antigen or RAMA [42], [43]. The authors proposed that lipid rafts concentrate the ROP complexes in vesicles budding from the Golgi. They further suggested that a transmembrane escorter in the vesicles could direct membrane trafficking in conjunction with cytoplasm trafficking machinery.

## DrpB and TgSORTLR Are Essential Players in *T. gondii* Apical Organelle Biogenesis

The basis for initial segregation of proteins from the parasite Golgi to the apical secretory organelles remained unclear until a large dynamin-like GTPase (DrpB) and a sortilin-like receptor (TgSORTLR) were recently described in the biogenesis of secretory organelles of *T. gondii* [44], [45]. Dynamins are large GTPases involved in numerous cellular processes including scission of vesicles by acting as mechano-enzymes or molecular switches. Conditional expression of a dominant negative mutant of DrpB, which resides in a cytoplasmic area close to the Golgi, led to absence of not only micronemes and rhoptries, but also dense granules [44]. Immunoelectron microscopic studies have not identified DprB associated with a well-defined membranous structure, and it has not been shown that the protein can bind to or transport MICs, RONs/ROPs, and dense granule proteins to their respective organelles. While it is not yet clear precisely how DrpB functions in vesicular traffic, its relationship to dynamin implies a role in vesicle fission from secretory or endosome-like organelles. Therefore, we speculate that DrpB plays a similar role as yeast VPS1, a dynamin-like protein that is essential for the formation of Golgi-derived vesicles destined for the yeast vacuole, a lysosome-like organelle. Interestingly, the phenotype of the yeast VPS1 mutant is analogous to the phenotype observed for DrpB, since in both cases the cargo molecules are misdirected to the constitutive secretory pathway.

Unlike other eukaryotes, *T. gondii* and other apicomplexans lack a mannose-6-phosphate receptor for sorting to the endosomal pathway, implying that they rely on an alternative mechanism. Sortilin, also known as VPS10 in yeast, is a type I single-pass transmembrane cargo receptor that functions in mannose-6-phosphate independent sorting to the endolysosomal system. Genetic ablation of TgSORTLR in *T. gondii* demonstrated that it is essential for the biogenesis of apical secretory organelles [45]. Parasites lacking TgSORTLR were completely devoid of micronemes and rhoptries. This study also showed that TgSORTLR is a type I transmembrane cargo receptor that localizes to Golgi and post-Golgi endosome-like compartments. The luminal portion of this receptor physically interacts with MICs and RONs/ROPs and regulates their transport to the apical secretory organelles. The phenotypic consequences of interfering with DrpB or TgSORTLR function are very similar, suggesting a function in the same step during vesicle formation and transport required for the biogenesis of apical secretory organelles. However, further studies are required to provide mechanistic insights of this linkage. Although the precise step that these proteins function in remains to be determined, it is possible that they contribute to vesicular budding from the trans-Golgi network or endosome-like organelles. In sum, our recent study reveals TgSORTLR as the only type I transmembrane cargo receptor identified so far in any apicomplexan parasite for its crucial roles in protein trafficking and biogenesis of secretory organelles.

## Merging the Endosomal and Exosomal Pathways for the Biogenesis of Apical Organelles

It is increasingly clear that the secretory pathway of apicomplexan parasites can be considered a stripped-down version of the more complicated machinery present in higher eukaryotes on both a functional and genomic basis (Figure 2). In contrast to mammalian cells and yeast, apicomplexan parasites are missing nearly all components of ESCRT machinery, with the exception of few marginal components whose functions remains to be determined (Figure 2). Several of the ESCRT protein complexes are conserved from archea to mammals, and are known to assemble into multi-subunit machinery that performs a topologically unique membrane bending and scission reaction into the lumen of multivesicular bodies, for example [46]. Although it remains possible that some components of the apicomplexan ESCRT machinery are present yet highly divergent, this scenario also implies a substantial alteration to the system. In addition to the absence or marked divergence of the ESCRT machinery, we also noticed that three important genes coding the ubiquitous coat Golgi-localized, gamma adaptin ear-containing, ARF-binding (GGA) proteins that regulate the trafficking of proteins between the trans-Golgi network and the lysosome are absent in apicomplexan genomes (Figure 2). Moreover, it is unlikely that other adaptors such as stonins and beta-arrestins participate in vesicle-mediated biogenesis of parasite organelles because they are also absent from or highly divergent in the genomes of *T. gondii* and other apicomplexans (Figure 2).

**Figure 3:**
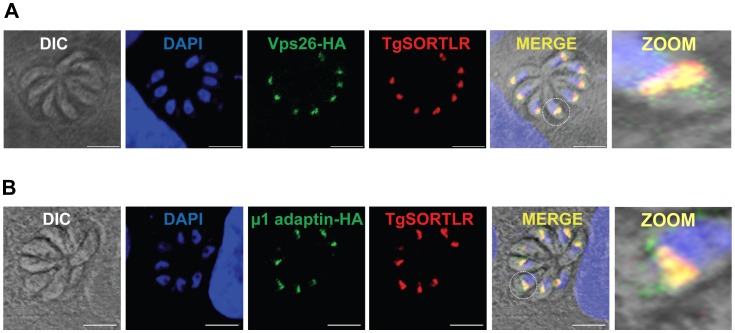
TgSORTLR co-localizes with TgVsp26 and Tgμ1-adpatin. (**A**) Confocal images of tachyzoites expressing endogenously tagged TgVps26-HA (green) that co-localizes with TgSORTLR (red). (**B**) Confocal images of tachyzoites expressing endogenously tagged Tgμ1adaptin-HA (red) and TgSORTLR (green). White circles indicate the zoomed areas showing co-distribution between TgSORTLR and Tgμ1adaptin-HA or TgVPS26-HA in the Golgi and post-Golgi. Scale bars, 5 µm.

**Figure 4:**
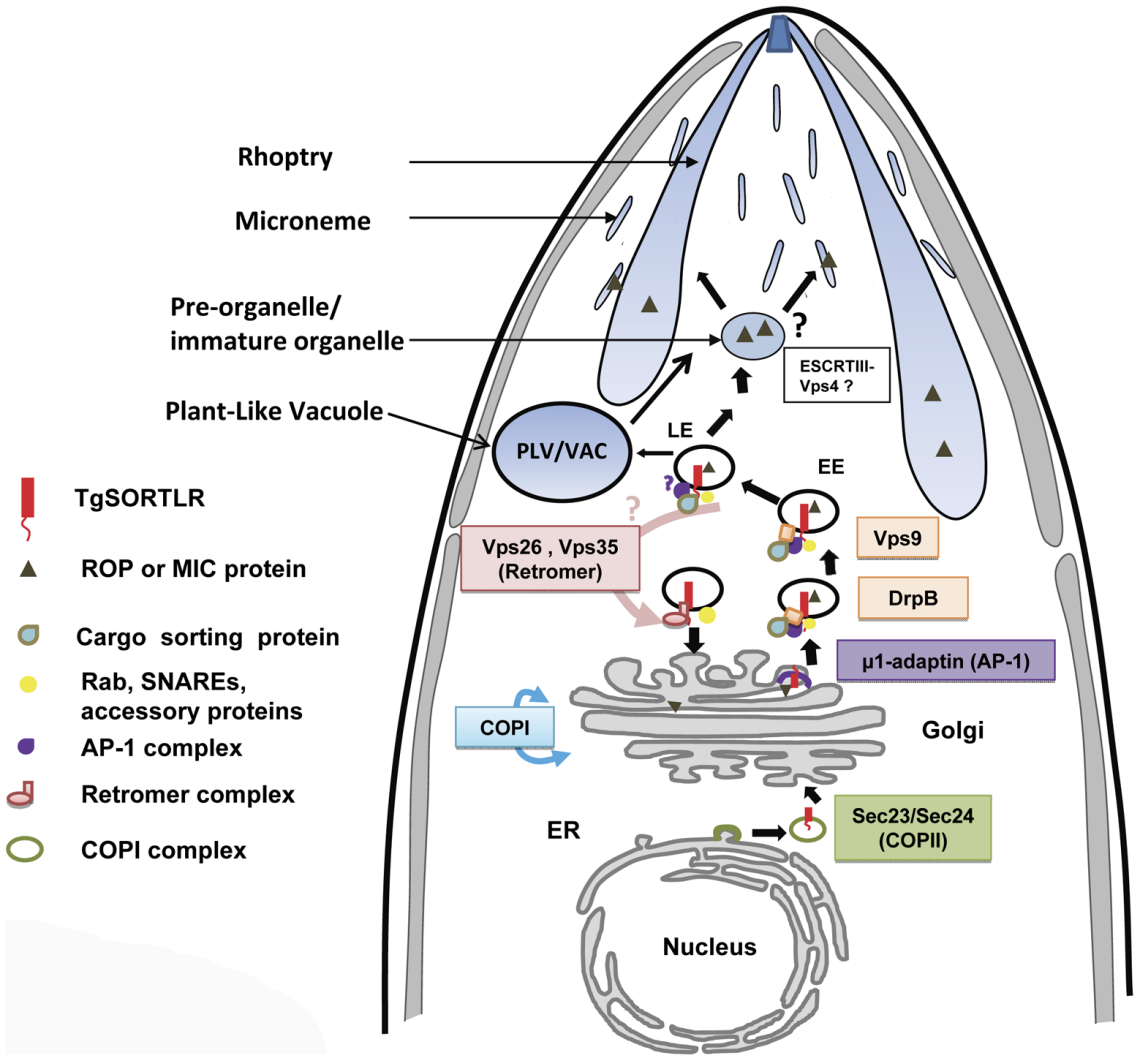
A model for TgSORTLR functions in protein sorting and the biogenesis of apical secretory organelles. We propose that TgSORTLR has a distinct role as a type I transmembrane cargo-protein receptor for ROPs and MICs of apicomplexan parasites. We observed TgSORTLR-positive structures that could be transport vesicles destined for the endolysosomal system or they might be integral to the endolysosomal system, i.e., early (EE) and late (LE) endosomes. The model further proposes that the cytoplasmic tail of TgSORTLR binds to AP-1, Sec23/24, clathrin, clathrin-associated adaptor protein, and VPS9, and this defines it as a key receptor involved in the anterograde transport of cargo ROP and MIC proteins. The binding of TgSORTLR to the retromer VPS26/VPS35 also indicates that this receptor is also involved in the retrograde transport of components. *T. gondii* lysosome-like, acidic vacuolar compartment (VAC), also termed the Plant-Like Vacuole (PLV), contains cathepsin proteases implicated in the proteolytic maturation of proproteins targeted to MICs. Proteolytic maturation likely occurs in the LE where conditions are thought to be more conducive for limited proteolysis.

One the other hand, other components of the anterograde and retrograde trafficking system are conserved in the Apicomplexa (Figure 2). Consistent with their functional importance, we recently confirmed that the C-terminal tail of TgSORTLR binds specifically to the cytosolic sorting complexes involved in anterograde transport or retrograde transport [45]. For anterograde transport, the TgSORTLR cytoplasmic tail not only binds clathrin and to three components of the AP1 and AP2 adaptor complexes, but also to homologues of Vps9 and of the COPII or coat complex transport proteins Sec23/Sec24 that ensure the directionality of anterograde membrane flow from the ER to the Golgi apparatus [47]. For retrograde transport, TgSORTLR binding to Vps26-Vps29-Vps35 indicated its association with the retromer complex, which mediates transport from endosomes to the trans-Golgi network. Epitope tagging several of these components including TgVps26 and the Tgμ1 adaptin (Figure 3A and 3B) revealed partial or full co-localization with TgSORTLR in the Golgi and post-Golgi compartments, supporting their role in trafficking between and possibly beyond these structures. This represents, first, a good validation of the possible functions of the endocytic components present in apicomplexan genomes (Figure 2) and, second, further evidence for the involvement of the endosome-like pathway for sorting to the apical secretory organelles (Figure 4).

Direct evidence that other endosomal trafficking factors are essential for the transport of ROPs and MICs came from a recent screen that identified Rab5A and Rab5C as crucial components [15]. Many eukaryotes utilize members of the Rab5 family for endocytosis of exogenous material and trafficking through EE. However, in *Toxoplasma*, overexpression of wild-type or mutant Rab5A or Rab5C resulted in the mistrafficking of ROPs and several MICs, possibly due to the saturation of Rab effector proteins that regulate vesicular trafficking. Intriguingly, this study also demonstrated that micronemes are organized into at least two independent subsets, adding another level of complexity to the parasite secretory system. These findings further support a model in which micronemes and rhoptries are tightly linked to a modified endosomal trafficking system, and that these apical secretory organelles might indeed correspond to types of secretory lysosomes seen, for example, in natural killer cells and platelets [40].

## Endocytosis: An Unresolved Enigma in Some Apicomplexan Parasites

While it is becoming clear that *Toxoplasma* is capable of using its endosome-like pathway for protein sorting and organelle biogenesis (Figure 4), several questions still remain concerning the ability of apicomplexan parasites to perform classical endocytosis mediated by surface receptors and vesicular coat proteins such as caveolin or clathrin. The machinery required for caveogenesis and function of caveolae in endocytosis and membrane trafficking is absent in the apicomplexan parasites, and no caveola-dependent invaginations have been seen in these parasites, so far [48]. Moreover, evidence of clathrin-dependent endocytosis by apicomplexan parasites is lacking. We recently localized the *Toxoplasma* clathrin heavy chain, TgCHC1, almost exclusively to the Golgi of the parasite and identified clathrin-coated vesicles. Knockdown of TgCHC1 as well as dominant negative expression of the TgCHC1-hub fragment demonstrated the important role of TgCHC1 in organization of the Golgi during replication and a complete block of protein transport to the IMC, micronemes, rhoptries, and dense granules. However, we were not able to identify any TgCHC1 at the parasite surface or at the micropore (also called a cytostome), which is considered a potential site of endocytosis (Figure 1A, labeled Mp). Also, extracellular *T. gondii* parasites are not capable of internalizing classical mammalian endocytic tracers such as fluorescent lipid dyes. Thus, while it is likely that TgCHC1 functions in Golgi and post-Golgi trafficking along with TgSORTLR and the AP1 complex and possibly the AP3 complex [49], it is less clear that TgCHC1 functions with surface receptors and the AP2 complex for classic endocytosis. The biological functions of the AP4 complexes expressed by apicomplexans (Figure 2) have not been investigated.

It is possible that apicomplexans use all of their AP complexes for intracellular trafficking of vesicles containing proteins destined to secretory organelles or the formation of the IMC. If so, this unique feature could be dictated by the intracellular lifestyle of the parasite living within a vacuole in mammalian cells where the acquisition of nutrients occurs by transport systems across the parasite plasma membrane. It should be noted, however, that *Plasmodium* uses its cytostome and endocytic system to conspicuously internalize and degrade hemoglobin and other proteins from the RBC cytosol, likely via a bulk flow mechanism. Hemoglobin degradation occurs within an acidic food vacuole that is thought to be the parasite equivalent of a lysosome. The extent to which *T. gondii* and other apicomplexan parasites use an analogous system of internalizing and digesting host-derived proteins while replicating in nucleated cells remains to be reported. This question has become particularly intriguing in light of the recent reports that *T. gondii* possesses a lysosome-like, acidic vacuolar compartment [34], [50], [51].

## Conclusions

Powerful reverse genetic strategies in *T. gondii* have established that DrpB, TgSORTLR, Rab5A, Rab5C, and CHC1 act as key trafficking components at Golgi cisternae and proximal vesicles for the biogenesis of apical secretory organelles. Disrupting these proteins leads to the absence of host cell egress, gliding motility, cell invasion, and *in vivo* infection because of the crucial roles for apical secretory organelles in these events. Follow-up studies will shed light on how these endosomal components are functionally orchestrated to ensure protein trafficking and organelle biogenesis. Despite its high conservation throughout the tree of life, the endosomal sorting receptor sortilin, for example, is not known to be essential in any other biological system. The essentiality of a sortilin-like receptor in *T. gondii* is probably due to a combination of lack of redundancy with a mannose-6-phosphate receptor and the key roles of the cargo since ROPs and MICs are both required for invading and surviving within the host cell. The first definitive genetic evidence of a unicellular eukaryote using its endosomal pathway as a conduit for proteins destined for regulated secretion suggests that the parasite has evolved a novel strategy of merging the endosomal and secretory systems to take advantage of endosomal proteases for activation of regulated secretory products. These discoveries lay the foundation for future work dissecting the mechanism of sortilin- or other endosome-based protein traffic. Understanding how these signals are organized into a hierarchy, are interpreted, and are regulated to mediate high-fidelity sorting into various intracellular compartments represents a major challenge in cell biology. Despite being touted as a multi-protein receptor, precisely how sortilin recognizes multiple cargo proteins remains poorly understood in any system. Investigating protozoan parasites, which possess fewer redundancies in sorting mechanisms than other eukaryotes and relatively simple early secretory structures (ER and Golgi), has the potential to yield additional fundamental insight into trafficking mechanisms. Efforts will focus on how each of the distinct secretory organelles are formed through the segregation and sorting of post-Golgi secretory vesicles devoted to the biogenesis of micronemes and rhoptries, two key organelles for parasite survival and pathogenesis. The findings to date provide a unique glimpse into a simplified yet elaborate sorting system in lower eukaryotes and offer exciting new opportunities to understand the specialized intracellular lifestyle of apicomplexan parasites.
